# Fast and robust Fourier ptychographic microscopy with position misalignment correction

**DOI:** 10.1117/1.JBO.28.11.116503

**Published:** 2023-11-28

**Authors:** Zicong Luo, Ruofei Wu, Hanbao Chen, Junrui Zhen, Mingdi Liu, Haiqi Zhang, Jiaxiong Luo, Dingan Han, Lisong Yan, Yanxiong Wu

**Affiliations:** a Foshan University, School of Physics and Optoelectronic Engineering, Foshan, China; b Huazhong University of Science and Technology, School of Optical and Electronic Information, Wuhan, China; c Ji Hua Laboratory, Foshan, China

**Keywords:** Fourier ptychographic microscopy, LED position misalignment correction, phase retrieval

## Abstract

**Significance:**

Fourier ptychographic microscopy (FPM) is a new, developing computational imaging technology. It can realize the quantitative phase imaging of a wide field of view and high-resolution (HR) simultaneously by means of multi-angle illumination via a light emitting diode (LED) array, combined with a phase recovery algorithm and the synthetic aperture principle. However, in the FPM reconstruction process, LED position misalignment affects the quality of the reconstructed image, and the reconstruction efficiency of the existing LED position correction algorithms needs to be improved.

**Aim:**

This study aims to improve the FPM correction method based on simulated annealing (SA) and proposes a position misalignment correction method (AA-C algorithm) using an improved phase recovery strategy.

**Approach:**

The spectrum function update strategy was optimized by adding an adaptive control factor, and the reconstruction efficiency of the algorithm was improved.

**Results:**

The experimental results show that the proposed method is effective and robust for position misalignment correction of LED arrays in FPM, and the convergence speed can be improved by 21.2% and 54.9% compared with SC-FPM and PC-FPM, respectively.

**Conclusions:**

These results can reduce the requirement of the FPM system for LED array accuracy and improve robustness.

## 1 Introduction

Optical microscopic imaging is an important technical tool in many fields, such as life sciences and biomedicine. Traditional optical microscopes are limited by the space-bandwidth product (SBP)^1^ of the optical imaging system and cannot simultaneously achieve a large field of view (FOV) and high-resolution (HR) imaging at the same time. Fourier ptychographic microscopy (FPM) is an emerging computational imaging technology that usually uses the combination of an optical microscope and a light emitting diode (LED) illumination array (programmable control) and combines phase recovery^2^^–^^4^ and the synthetic aperture principle^5^^–^^8^ to restore the HR intensity and phase image of the sample. To a certain extent, it overcomes the limitations of traditional optical microscopes of the difficulty they face in balancing a large FOV and HR imaging, and thus constitutes an improvement over traditional optical microscopy. In contrast to traditional optical microscopes, FPM transfers the high-frequency information in an object that initially surpasses the system cutoff frequency to within the passband of the system through multi-angle illumination,^9^ collects a series of low-resolution (LR) images at the camera side, and then uses a phase recovery algorithm to stitch them in the frequency domain to achieve large SBP and quantitative phase imaging.^10^ The summation of the numerical aperture of the objective (${\mathrm{NA}}_{\mathrm{obj}}$) and the numerical aperture of illumination (${\mathrm{NA}}_{\mathrm{illu}}$) determines the reconstruction resolution.^11^ Simultaneously achieving a large FOV and HR imaging has significance in microscopic imaging. Therefore, FPM-related theories and technologies have been widely researched and applied in the fields of digital microscopy and life sciences.^12^^–^^15^

In an FPM system, LEDs at different positions on the LED array generate plane waves with different incident angles to illuminate the sample. The spatial position of each LED directly determines the position of the corresponding LR image in the frequency domain;^16^ that is, each LED corresponds to a sub-aperture in the frequency domain. However, in the process of production and assembly, there will inevitably be a certain position error in the LED array, whereupon the collected light intensity information and the ideal position information of the LEDs used in the reconstruction process cannot completely correspond, which transmits incorrect information into the captured LR image and affects subsequent reconstruction. When a certain offset exists in the LED array position, artifacts and wrinkles occur in the reconstructed image, leading to a decline in the quality of the reconstructed image. Therefore, the study of LED position misalignment correction is of great significance for improving the quality of reconstructed images. Position-correction methods have been proposed to solve this problem. In 2016, Sun et al. proposed PC-FPM based on the simulated annealing (SA) and non-linear regression technology, which can correct the LED position errors, and also introduced the LED array global position misalignment model;^17^ however, the speed still needs to be improved. Pan et al. proposed an SC-FPM based on the SA algorithm, LED intensity correction, and an adaptive step-size strategy to correct the system mixing error and improve the robustness of the FPM to the system mixing error.^18^ In 2018, Chen et al. proposed the rpcFPM, which uses feedback parameters and objective function constraints to correct the random position error of each LED and obtain good initial guesses;^19^ however, the performance of this algorithm is limited by the ePIE algorithm^20^ and may only reach local optima. Zhou et al. also proposed an mcFPM method based on a spatial domain search using the SA algorithm, which isolates the update of the misalignment parameters from the FPM iteration and uses the global position misalignment model to correct the global offsets;^21^ however, it cannot correct the height factor, and when there are many parameters, the accuracy and speed of global convergence are not sufficient. Zhao et al. proposed a trainable neural network for position correction and image reconstruction using the real and imaginary parts and the different position errors of each aperture as the weights of the convolution layer to achieve the correction of the aperture position deviation;^22^ however, this method is still relatively time-consuming. Therefore, it is necessary to propose a faster convergence speed and a more robust performance for the LED position misalignment correction algorithm, which will provide a guide for the future direction of our work.

In our previous study, we improved the conventional FPM reconstruction algorithm, which is referred to as the AA algorithm.^23^ The AA algorithm improved the rate of convergence and robustness to noise of the reconstruction algorithm; however, it does not consider the influence of LED position misalignment in the FPM system on the reconstructed image. Therefore, this study improves the FPM position correction method based on the SA algorithm and proposes a position misalignment correction method (AA-C) with an improved phase recovery strategy that optimizes the spectral function update strategy by calculating the threshold and introducing an adaptive control factor while simultaneously employing the position correction method, which can converge faster and obtain better reconstruction results than the traditional correction method. In this study, simulations and real experiments were conducted to demonstrate the effectiveness and robustness of the improved method for LED position misalignment correction, which not only speeds up the rate of convergence but also reduces the adverse impact of LED position misalignment on image reconstruction, reduces the positioning accuracy requirements for LED arrays in FPM systems, and improves the robustness of the FPM system.

The remainder of this paper is organized as follows. We introduce the basic model of the FPM and LED array position model in Sec. 2.1. Then, we introduce the flow and specific steps of the proposed AA-C algorithm in Sec. 2.2. We verify the effectiveness of the AA-C algorithm by simulations and practical experiments in Sec. 3. Finally, we summarize the conclusions in Sec. 4.

## 2 Principles and Methods

### 2.1 FPM Forward Imaging and LED Position Misalignment Model

**Fig. 1:**
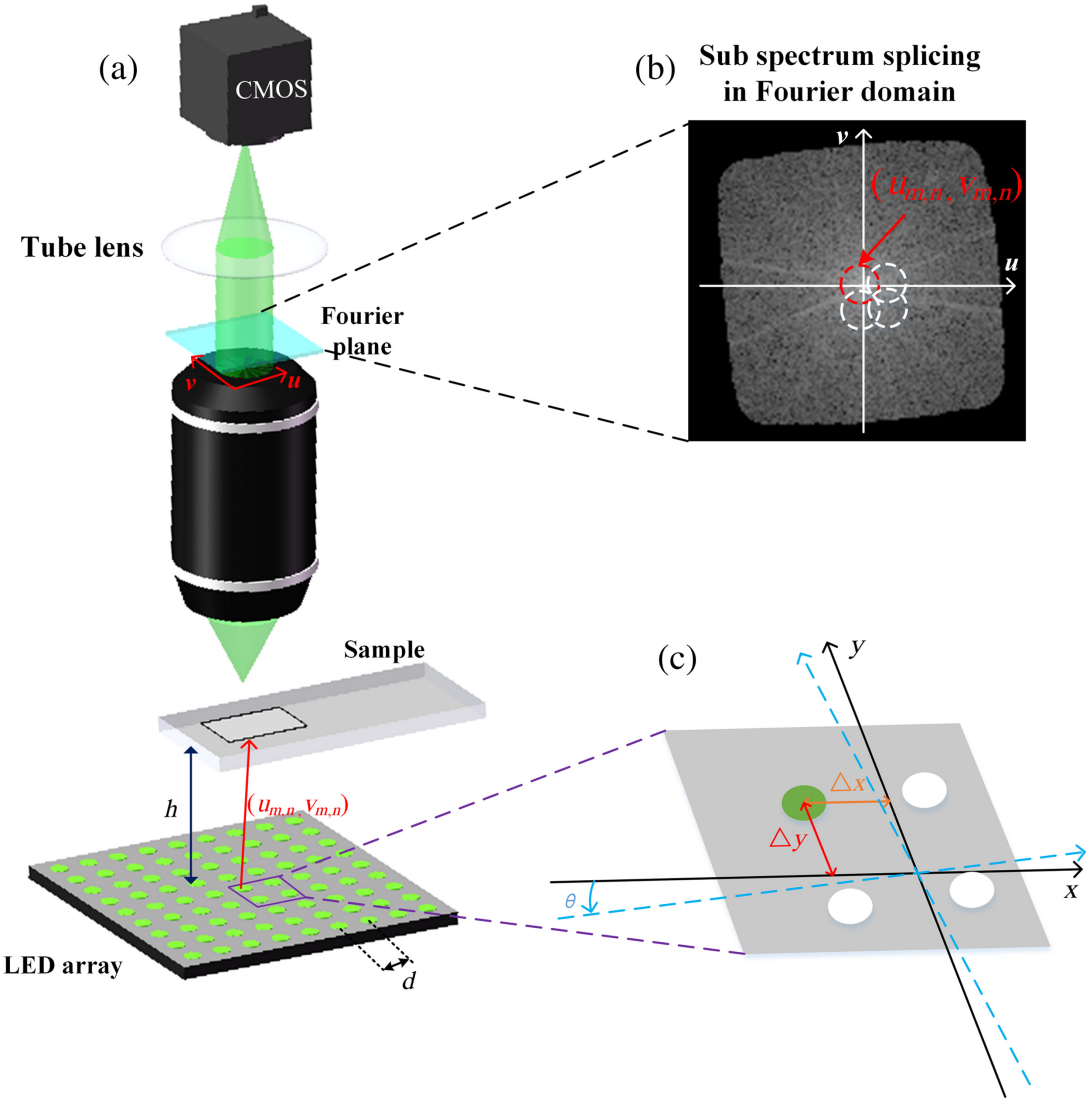
FPM model and imaging process and schematic diagram of LED position misalignment. (a) FPM forward imaging model. (b) Schematic diagram of sub-spectrum information splicing in Fourier domain. (c) Schematic diagram of LED position misalignment. The black solid line coordinate axis is the ideal coordinate axis, the blue dashed line is the actual coordinate axis, and the green LED is the center LED of the LED array.

Figure 1(a) shows an FPM forward imaging model. A typical FPM model mainly includes an LED array, samples, a low-NA objective lens, and a camera. The FPM imaging process included two parts. One is front-end images acquisition, and the other part is back-end data reconstruction. In images acquisition, different angles of plane wave illumination are provided by sequentially lighting the LEDs. The camera is used at the imaging end to capture the corresponding LR images, these images captured will be used for image reconstruction. The dotted circles in Fig. 1(b) represent the sub-spectrum information corresponding to the sample under the illumination of plane waves at different angles.

First, we establish a position model for the LED array. As shown in Fig. 1(a), the LED array is placed on a plane perpendicular to the optical axis, and the distance between neighboring LEDs in the array is regarded as the same. Ideally, the parameters in FPM are accurate; however, in practice, these parameters are biased. Four position factors are defined to determine the position of each LED element.^17^ The schematic diagram of LED position misalignment is shown in Fig. 1(c), including the rotation factor θ, the position deviation factors Δ x, Δ y along the x axis and y axis, and the perpendicular distance h from the LED array to the sample, and then the position of each LED element can be expressed as

$$[\begin{array}{cc} x_{m,n} & y_{m,n} \end{array}]=[\begin{array}{cc} md_{\mathrm{LED}} & nd_{\mathrm{LED}} \end{array}]\cdot [\begin{array}{cc} \cos\;\theta & -\sin\;\theta \\ \sin\;\theta & \cos\;\theta \end{array}]+[\begin{array}{cc} \Delta x & \Delta y \end{array}],$$ (1)

where $x_{m,n}$, $y_{m,n}$ represent the positions of the LED elements on row m and column n, and $d_{\mathrm{LED}}$ is the distance between adjacent LEDs. For a subregion of the sample, the illuminated wave vector $\left(u_{m,n},v_{m,n}\right)$ from ${\mathrm{LED}}_{m,n}$ can be expressed as

$$u_{m,n}=k\frac{x_{o}-x_{m,n}}{\sqrt{{\left(x_{o}-x_{m,n}\right)}^{2}+{\left(y_{o}-y_{m,n}\right)}^{2}+h^{2}}},\;v_{m,n}=k\frac{y_{o}-y_{m,n}}{\sqrt{{\left(x_{o}-x_{m,n}\right)}^{2}+{\left(y_{o}-y_{m,n}\right)}^{2}+h^{2}}},$$ (2)

where $k=\frac{2\pi }{\lambda }$, λ is the center wavelength of LEDs, $\left(x_{o},y_{o}\right)$ is the position coordinate of the center of the reconstructed small region. When plane wave with wave vector $\left(u_{m,n},v_{m,n}\right)$ from ${\mathrm{LED}}_{m,n}$ irradiates the sample, the spectrum of the complex amplitude of the light wave field on the camera-receiving surface can be expressed as

$$\phi_{m,n}(u,v)=O(u-u_{m,n},v-v_{m,n})\cdot P(u,v),$$ (3)

where O(u,v) is the HR spectrum of object function and P(u,v) represents the pupil function of the objective, which can be considered a low-pass filter. It is 1 in the passband and 0 outside the passband, and (u,v) is the frequency domain coordinate. The obtained intensity image can be expressed using the following equation:

$$I_{m,n}(x,y)={\left|F^{-1}[\phi_{m,n}(u,v)]\right|}^{2}.$$ (4)

Here, $F^{-1}$ expresses the Fourier inverse transform. In the images’ reconstruction process, the LR images captured above constitute the amplitude constraints of the FPM in the spatial domain, and P(u,v) is used as a spectrum support domain constraint in the frequency domain. FPM stitches the LR images collected under different illumination angles in the frequency domain and obtains the HR complex amplitude result of the object through iterative convergence.

### 2.2 Flow of the Proposed Algorithm

**Fig. 2:**
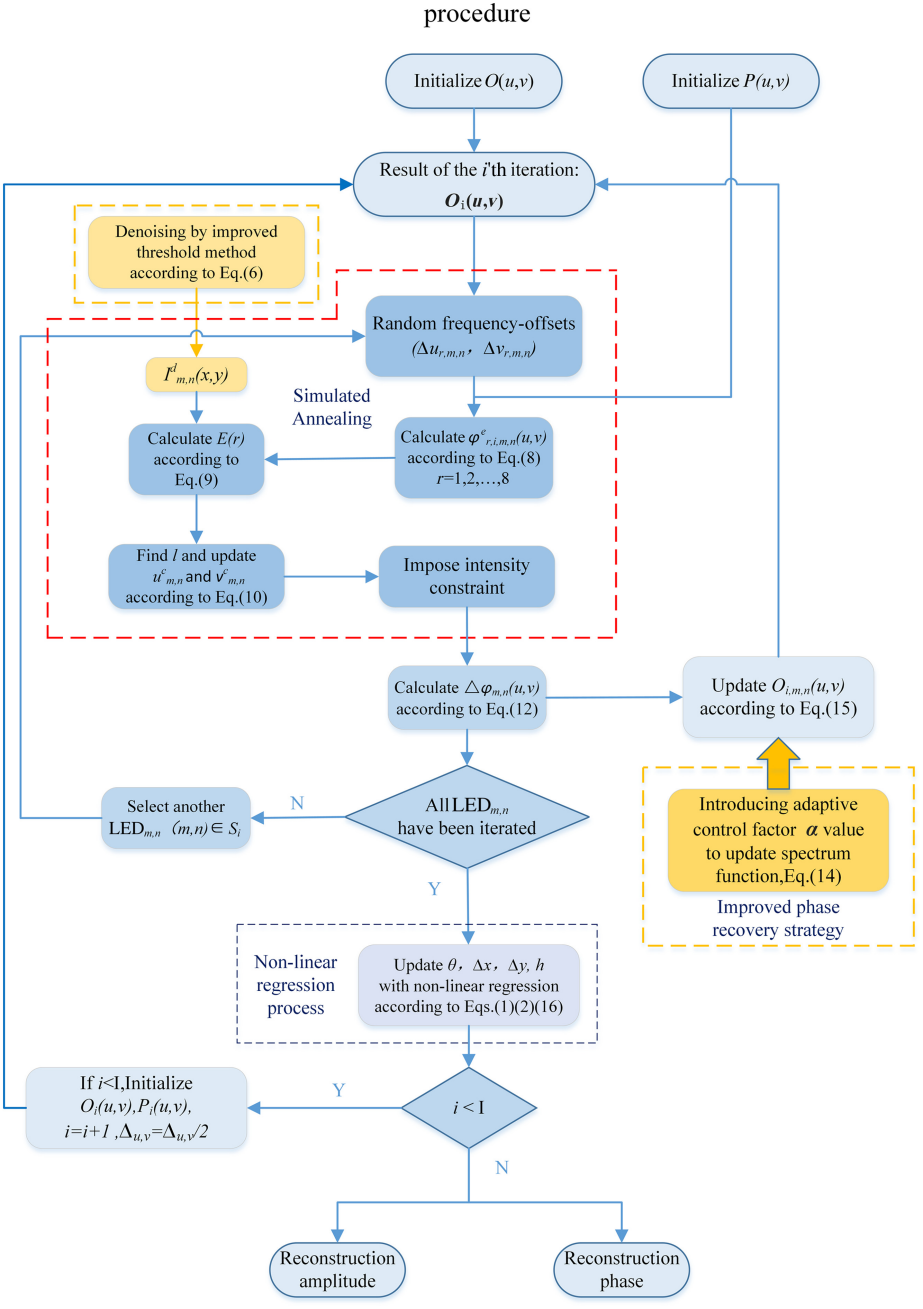
Flowchart of the AA-C algorithm.

A flowchart of the proposed method is shown in Fig. 2. The reconstruction algorithm is mainly divided into four modules: (1) The improved threshold denoising module. (2) The simulated annealing module. In the reconstruction process, the optimal solution of the frequency aperture is searched for subsequent spectrum updating. (3) The improved phase recovery strategy module. The update of the spectrum function is optimized by introducing an adaptive control factor. (4) Non-linear regression module. It is used to fit and estimate the global position factor to avoid confusion and disorder in the LED position obtained by the correction.

The following are the specific steps and process of algorithm implementation:

Step 1: Initialization: Before running the iterative algorithm, take the corresponding LR image illuminated by the central LED and perform Fourier transform to obtain the initial HR spectrum guess O(u,v) of the sample. The pupil function is P(u,v), which remains constant during the iterative update. $O_{i}(u,v)$ is recorded as the sample spectrum of the i’th iteration.
Step 2: Use the pupil function to intercept the information in the initial HR spectrum of the sample, which is equivalent to low-pass filtering, and then inverse Fourier transformed to the spatial domain to generate the target complex amplitude image $\sqrt{I_{m,n}^{t}}\;\exp(i\varphi_{m,n}^{t})$, where t denotes the target image. Noise reduction is then performed by calculating the noise threshold, defined as the difference in the arithmetic mean between the image actually obtained and the target image:^24^
  $${\text{Threshold}}_{m,n}=\langle I_{m,n}(x,y)\rangle -\langle I_{m,n}^{t}(x,y)\rangle ,$$ (5)
  where $I_{m,n}(x,y)$ and $I_{m,n}^{t}(x,y)$ represent the actually acquired image and target image, respectively. The noise threshold calculated using Eq. (5) is then subtracted from the actually acquired image for noise reduction:
  $$I_{m,n}^{d}(x,y)=I_{m,n}(x,y)-{\text{Threshold}}_{m,n},$$ (6)
  where $I_{m,n}^{d}(x,y)$ represents the denoising image, which is used to update the amplitude information of the LR estimated light field in step 4.
Step 3: In each iteration, the angle corresponding to each LED is processed (i.e., the LR image corresponding to each LED is updated) before completing one iteration of the algorithm. However, in the initial iteration, we generally use bright-field (BF) images because they contain relatively more low-frequency information, which is more important for image reconstruction; BF images are less affected by noise, and more accurate parameters can thus be obtained at the beginning. Therefore, we first iterate over the images with low ${\mathrm{NA}}_{\mathrm{illu}}$ to correct the position of the low-frequency aperture in the frequency domain. For example, in the simulation, we used an 11×11 LED array and repeated the LR images corresponding to the middle 5×5 LEDs in the first 10 iterations. $O_{i}(u,v)$ must be initialized at the end of each initial iteration. After 10 initial iterations, 121 images (i.e., all captured images) in subsequent iterations must be used for iteration for more accurate position correction. We define the LED update range $S_{i}$ as
  $$S_{i}=\{\begin{array}{ll} \left\{(m,n)|m=-2,\ldots ,2,n=-2,\ldots ,2\right\} & i\leq 10\\ \left\{(m,n)|m=-5,\ldots ,5,n=-5,\ldots ,5\right\} & \text{else} \end{array}.$$ (7)

Then, in the FPM reconstruction process, the SA algorithm^25^ is used to search in the frequency domain to find the optimal solution for the frequency aperture and correct the incident wave vector of the LED. From Eq. (2), it can be identified that the incident wave vector $\left(u_{m,n,}v_{m,n}\right)$ is related to the position of the LED elements $\left(x_{m,n},y_{m,n}\right)$ and the distance h from the LED array to the sample. In addition, from Eq. (1), it can be observed that the position of LED elements $\left(x_{m,n,}y_{m,n}\right)$ depends on the rotation factor θ, the positional deviation Δx, Δy along the x axis and the y axis. Therefore, searching for the frequency aperture optimal solution is in fact, searching for the optimal solution for the LED’s positional deviation factor (θ,Δx,Δy,h). First, we calculate a further estimation of a group of frequency apertures $\varphi_{r,i,m,n}^{e}(u,v)$ (r∈{1,2,…,8}, representing eight different frequency shift directions, i.e., a random search in eight directions), each of which has a random frequency offset $\left(\Delta u_{r,m,n},\Delta v_{r,m,n}\right)$. Here, we set the search step $\Delta_{u,v\;}=6$ for SA, which is our predefined value, which gradually decreases with increasing iteration time. The r’th estimate of the frequency aperture is given as

$$\varphi_{r,i,m,n}^{e}(u,v)=O_{i}(u-(u_{m,n}+\Delta u_{r,m,n}),v-(v_{m,n}+\Delta v_{r,m,n}))P(u,v).$$ (8)

The simulated target complex amplitude image is then: $\phi_{r,i,m,n}^{e}(x,y)=F^{-1}\{\varphi_{r,i,m,n}^{e}(u,v)\}$, and the intensity image is: $I_{m,n}^{e}(x,y)={\left|\phi_{r,i,m,n}^{e}(x,y)\right|}^{2}$. Then, each target intensity image is compared with the actual image (after noise reduction), the difference is calculated, and the error measure is defined as

$$E(r)=\underset{x,y}{\sum }{\left(I_{m,n}^{e}(x,y)-I_{m,n}^{d}(x,y)\right)}^{2}.$$ (9)

A smaller value of E(r) indicates that the intensity distribution of the simulated target image is closer to the actual acquired image; that is, the error is smaller. Find the LED frequency-domain coordinate offset corresponding to the minimum light intensity error (i.e., the minimum E(r)) of the LR light intensity images, mark the index of the minimum E(r) as l, and update the position of the frequency aperture as follows.

$$l=\arg\;\min[E(r)]\;u_{m,n}^{c}=u_{m,n}+\Delta u_{l,m,n}\;v_{m,n}^{c}=v_{m,n}+\Delta v_{l,m,n}.$$ (10)

Step 4: After updating the position of the frequency aperture $\left(u_{m,n}^{c},v_{m,n}^{c}\right)$, impose intensity constraints on the actually captured images, replace the target complex amplitude information with the LR image data after denoising in step 2, keep the phase information unaltered, and update the LR target image as follows:
  $$\phi_{m,n}^{\prime }(x,y)=\sqrt{I_{m,n}^{d}(x,y)}\frac{\phi_{l,i,m,n}^{e}(x,y)}{\left|\phi_{l,i,m,n}^{e}(x,y)\right|}.$$ (11)

A Fourier transform is then performed on the updated LR target image to obtain the updated HR spectrum after replacing the amplitude: $\varphi_{m,n}^{\prime }(u,v)=F\{\phi_{m,n}^{\prime }(x,y)\}$. Calculate the difference between the estimated light field in the frequency domain and the updated estimated light field:

$$\Delta \varphi_{m,n}(u,v)=\varphi_{m,n}^{\prime }(u,v)-\varphi_{l,i,m,n}^{e}(u,v).$$ (12)

Step 5: To reduce the effect of abrupt changes in the value of the pupil function on the spectrum update, the ratio is added to the spectrum update process, defined as
  $$W=\frac{\left|P_{m,n}(u,v)\right|}{{\left|P_{m,n}(u,v)\right|}_{\max}}.$$ (13)

Then, the adaptive control factor α is added to optimize the updating procedure of the target spectrum function to improve the reconstructed image quality and convergence speed. The value of α defined as

$$\alpha =2-\langle \sum {\text{Threshold}}_{m,n}\rangle .$$ (14)

The value of α is related to the noise reduction threshold. The target spectrum function used for the update after optimization is shown below:

$$O_{i+1}(u-u_{m,n},v-v_{m,n})=O_{i}(u-u_{m,n}^{c},v-v_{m,n}^{c})+(\alpha -W)\frac{P_{i}^{*}(u,v)}{{\left|P_{i}(u,v)\right|}^{2}+\delta_{1}}\Delta \varphi_{m,n}(u,v),$$ (15)

where “*” represents the complex conjugate operation, $\delta_{1}$ is a regularization constant used to maintain numerical stability and prevent the denominator from becoming zero. We usually set $\delta_{1}=1$.

Step 6: Repeat steps 2 to 5 for different LEDs on the LED array until all spectral information within all sub-apertures have been updated (i.e., LR images corresponding to all LEDs in the $S_{i}$ range are used for the update), which means that one iteration is completed.

We then use the non-linear regression algorithm^16^ to update the four global factors of the LED array position (θ,Δx,Δy,h). The process can be represented as

$$Q(\theta ,\Delta x,\Delta y,h)=\underset{m,n}{\sum }[{\left(u_{m,n}(\theta ,\Delta x,\Delta y,h)-u_{m,n}^{c}\right)}^{2}+{\left(\nu_{m,n}(\theta ,\Delta x,\Delta y,h)-\nu_{m,n}^{c}\right)}^{2}],\;(\theta ,\Delta x,\Delta y,h{)}^{c}=\mathrm{argmin}[Q(\theta ,\Delta x,\Delta y,h)].$$ (16)

Here, the problem of LED array position misalignment correction can be regarded as the position misalignment factors (θ,Δx,Δy,h) for each LED to find the optimal solution, where Q(θ,Δx,Δy,h) is used to represent the error between the theoretical value and the corrected value of the central coordinate of the sub-aperture in the frequency domain. We update the global position factors through the non-linear regression process and solve the position factors ${\left(\theta ,\Delta x,\Delta y,h\right)}^{c}$ corresponding to the minimum Q(θ,Δx,Δy,h).

Step 7: Repeat steps 2 to 6 to obtain the updated global position factors, from which the frequency domain coordinates of the LED are updated to correct for LED position misalignment, until the iterative algorithm converges or the set number of iterations is reached so that finally completes the reconstruction process.

## 3 Experiments and Results

### 3.1 Simulation Experiment

To verify the effectiveness of the proposed algorithm, simulation validation was first conducted. In the simulation, the center 11×11 LEDs were selected to provide angle-changing illumination at a position of 56.1 mm beneath the sample, the distance between adjacent LEDs is 4 mm, the center wavelength of the LEDs is 531 nm, and the objective is 4×0.1  NA. The pixel size of the camera is 2.4  μm×2.4  μm. The “Cameraman” and “Westconcordorthophoto” (384×384  pixels) are initial input HR intensity image and phase image, respectively. In the simulation, we introduce four position factors (θ,Δx,Δy,h) into the original image.

**Fig. 3:**
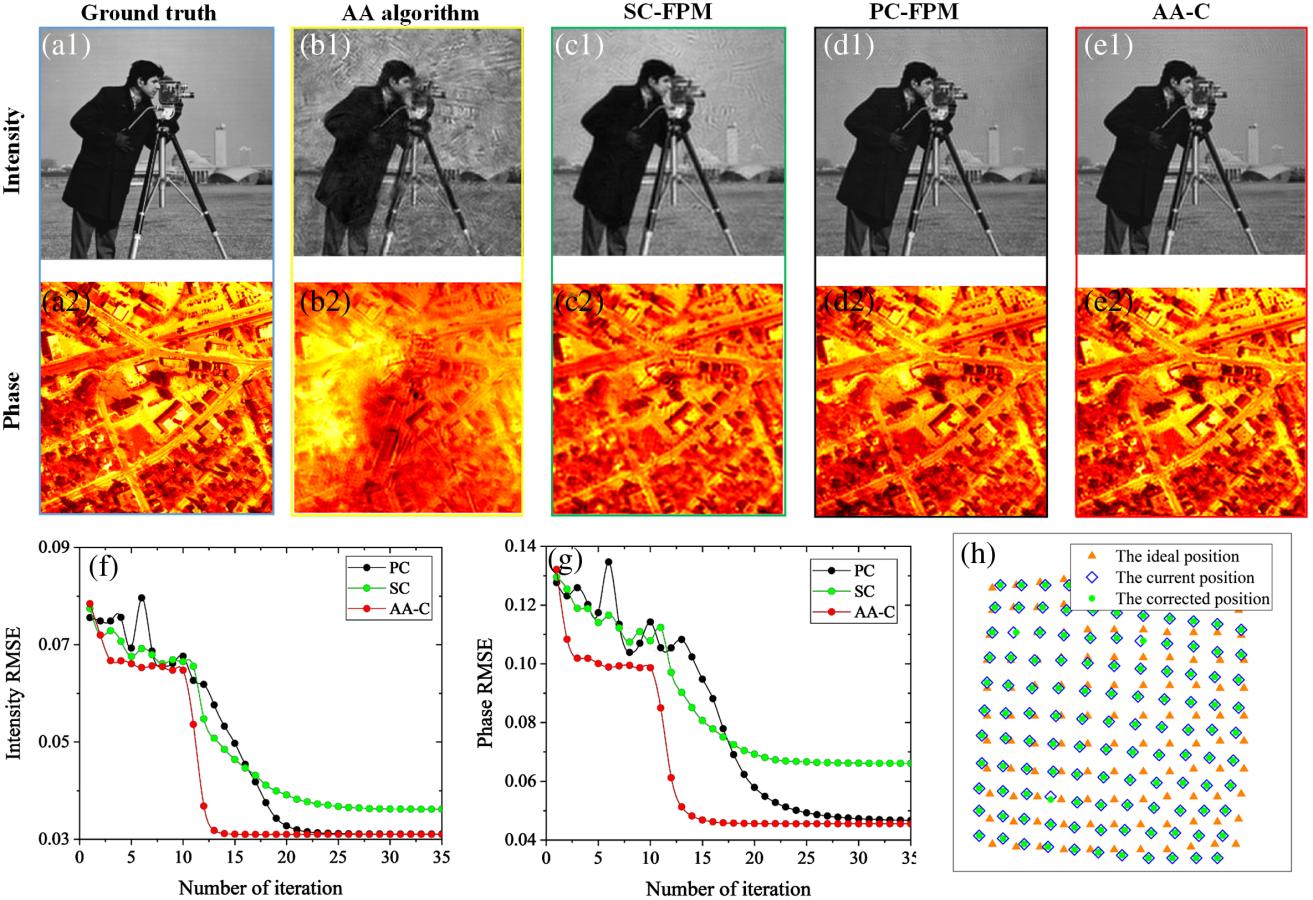
Comparison of simulation reconstruction results of different algorithms. (a1) and (a2) Simulation of the input HR intensity and phase distribution for complex samples. (b)–(e) Reconstruction results of the AA algorithm, SC-FPM, PC-FPM, and AA-C algorithm. (f) and (g) RMSE curves of the HR intensity and phase images reconstructed by the SC-FPM, PC-FPM, and AA-C algorithm with the iterative conditions. (h) The position schematic of the frequency aperture.

We set the range of the introduced position misalignment parameters as: θ∈[−5°,5°], Δx∈[−1000  μm,1000  μm], Δy∈[−1000  μm,1000  μm], Δh∈[−1000  μm,1000  μm], because once these four factors move out of this range, the position error will be obvious. At this point, the LED array can be aligned through the initial calibration of the physical position. Here, we set the position misalignment parameters to θ=5°, Δx=1000  μm, Δy=1000  μm, and Δh=1000  μm. In total, 121 LR images were obtained from the simulation, and the overlap rate in the frequency domain was 55.62%. Subsequently, in the image reconstruction process, the ideal position of the LED is used for iterative reconstruction. We used and compared four algorithms. Figure 3 shows the simulation results. Figures 3(a1) and 3(a2) show the input HR intensity and phase distributions of the simulated complex samples, respectively, whereas Figs. 3(b1) and 3(b2) show the reconstruction results of the AA algorithm without correcting for position misalignment. Owing to the lack of correction for position misalignment errors, the reconstructed intensity and phase of the image have artifacts and wrinkles in the background, seriously affecting the clarity of the image. The results of the SC-FPM reconstruction are shown in Figs. 3(c1) and 3(c2), the quality of the reconstructed images is significantly improved, but there remain some artifacts in the reconstructed images. In comparison, the reconstructed image quality of the PC-FPM is improved, but there remain a few artifacts in the background, as shown in Figs. 3(d1) and 3(d2). Finally, the method presented in this paper is shown in Figs. 3(e1) and 3(e2), which shows that the reconstruction results have better background uniformity and fewer artifacts than other algorithms, indicating better correction of the position misalignment and better reduction of the impact of the position misalignment on reconstruction quality.

The root-mean-square error (RMSE) curves of the HR intensity image and the phase image reconstructed by the SC-FPM, PC-FPM, and AA-C algorithms are shown in Figs. 3(f) and 3(g). It can be seen from the RMSE curve that the AA-C algorithm converges faster, the value after iterative stable convergence is smaller, and the reconstruction results are better. Figure 3(h) shows the position schematic of the frequency aperture, where the orange triangle represents the ideal position of the LED, the blue diamond represents the current position of the LED, and the green circle represents the position of the LED after correction using the AA-C algorithm. It can be seen that almost all the green circles coincide with the blue diamond, indicating that the frequency aperture positions are well corrected, illustrating the effectiveness of the algorithm in correcting the LED array position.

Table 1 also shows the peak signal-to-noise ratio (PSNR) and convergence time of the reconstructed images using different algorithms. The reconstruction intensity and phase PSNR value of the AA-C algorithm are higher than those of the other algorithms in the table, and the convergence time is the shortest. AA-C converged in 17 iterations, and convergence required 25 and 29 iterations for SC-FPM and PC-FPM, respectively. The convergence speed is 21.2% faster than that of SC-FPM and 54.9% faster than that of PC-FPM. The results indicate that the AA-C algorithm reconstructs images with better quality and faster convergence.

**Table 1:** PSNR values and convergence time of reconstruction intensity and phase for different algorithms in the case of iterative stabilization. Bold characters represent the indicators of the proposed method that are better.

Algorithms	Reconstructed intensity	Reconstructed phase	Time (s)	Iteration
	PSNR (dB)			
AA	22.78	12.23	—	—
SC-FPM	27.31	22.47	15.585	25
PC-FPM	29.72	24.60	26.927	29
AA-C	**30.22**	**26.59**	**11.097**	**17**

### 3.2 Real Experiment

The simulation results verified the effectiveness of the AA-C algorithm. To further verify the performance of the algorithm, we tested it using image data collected from actual experiments. The USAF resolution target was selected as a sample, and the experimental parameters were consistent with those used in Sec. 3.1. At the beginning of the experiment, precise mechanical correction of the LED array position was not performed. In the experiment, we captured 121 LR images to perform FPM reconstruction.

**Fig. 4:**
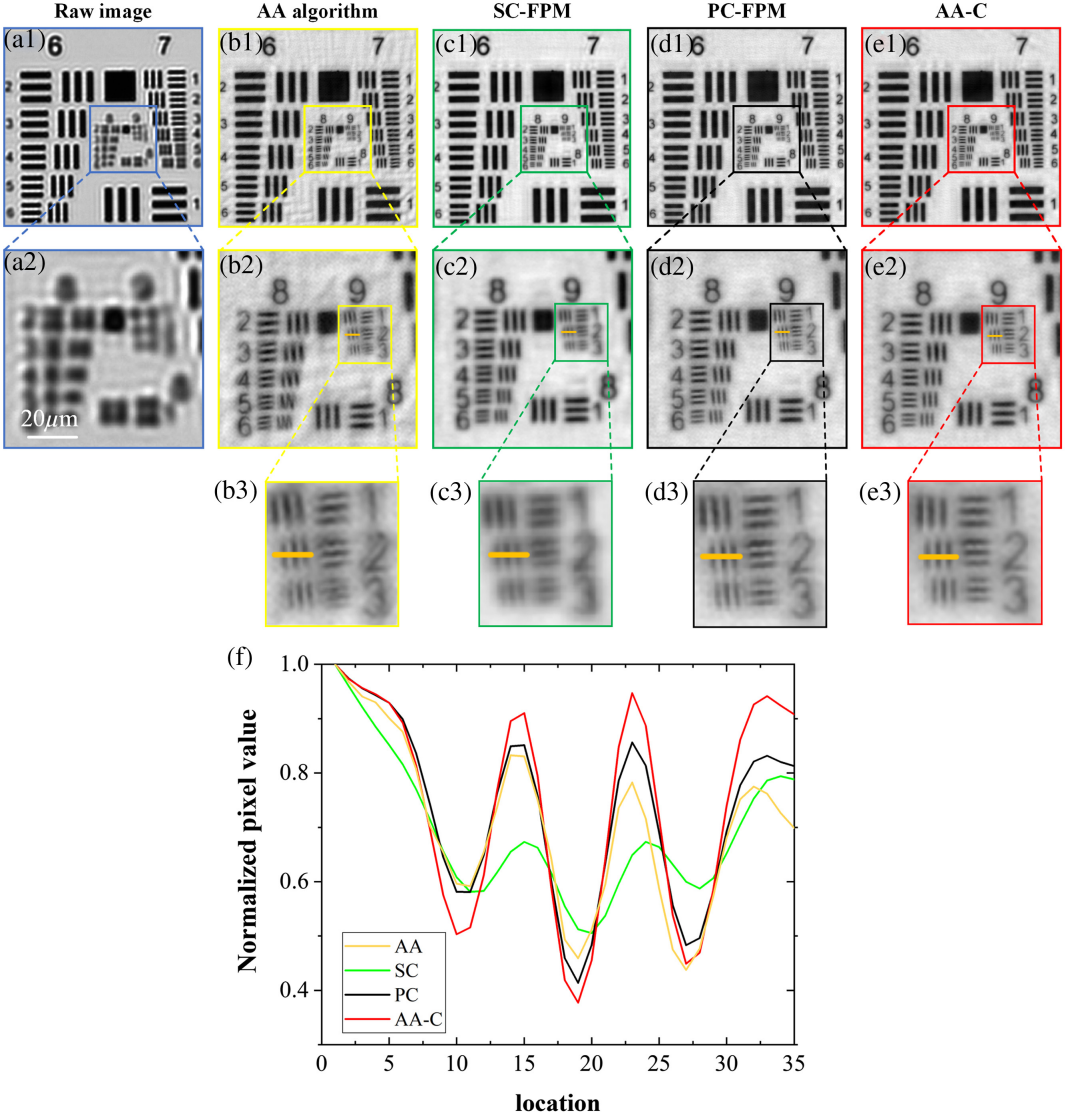
USAF 1951 resolution target experiment. (a1), (a2) LR images taken at the central FOV. (b1)–(b3) Reconstruction results of the AA algorithm. (c1)–(c3) Reconstruction results of the SC-FPM algorithm. (d1)–(d3) Reconstruction results of the PC-FPM algorithm. (e1)–(e3) Reconstruction results of the AA-C algorithm. (f) Pixel normalized intensity curve.

Figure 4 shows the reconstruction results for the different algorithms. Figures 4(a1) and 4(a2) show the LR image (400×400  pixels) captured in the central FOV and its enlarged view, respectively. For the AA algorithm, the reconstructed image shows severe artifacts and folds owing to the lack of position misalignment correction, as shown in Figs. 4(b1)–4(b3). The results of the SC-FPM reconstruction are shown in Figs. 4(c1)–4(c3). The background uniformity of the reconstructed image has been greatly improved and the artifacts and folds are much reduced. However, there is a loss of resolution. After zooming in, it can be seen that the ninth group of line-pair elements is slightly blurred and not clearly distinguished. Figures 4(d1)–4(d3) show the reconstruction results of the PC-FPM. Compared with Figs. 4(c1)–4(c3), the resolution is better, but the eighth group of line pairs appears to be tilted and not sufficiently straight. Figures 4(e1)–4(e3) show the reconstruction results of the AA-C algorithm. It can be seen that each group of line pairs on the resolution board can be clearly distinguished, the background is clear, and the line pairs have not been distorted or deformed. Overall, the reconstruction effect of the AA-C algorithm is the best. In addition, to further quantify and evaluate the reconstruction results, the trends of the pixel-normalized intensity value curves of the marked delineated areas in Figs. 4(b3)–4(e3) were compared, as shown in Fig. 4(f). An analysis of the curves shows that the red curve traces have the highest contrast, indicating that the images reconstructed by the AA-C algorithm have higher contrast and clearer details.

To further validate the effectiveness of the algorithm, we experimented with biological samples. Select human tumor cells were selected as a sample, and an 11×11 LED array was used to provide angle-change illumination. The other experimental parameters were consistent with the USAF 1951 resolution target experiment in Sec. 3.2. In all, 121 LR images were captured, and the collected images of human tumor cells were reconstructed and restored. Refractive indices of biological samples can be reflected by phase information. Compared to the image amplitude, the influence of the error on the phase information is generally greater.^26^

**Fig. 5:**
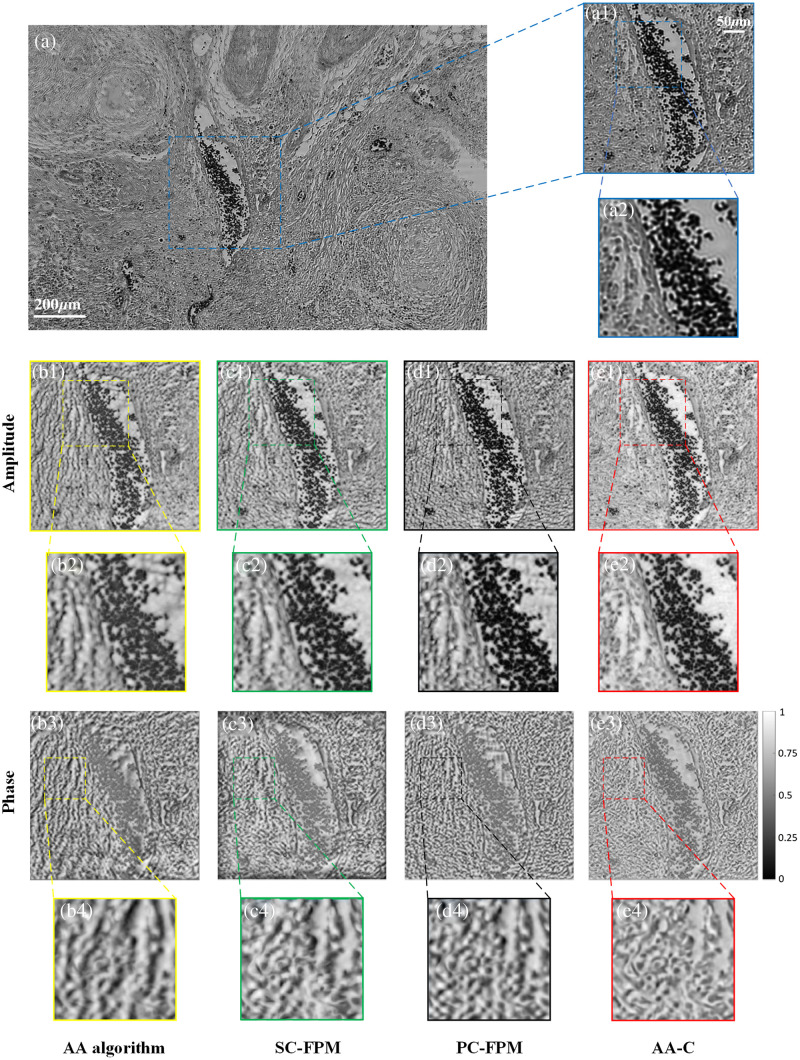
Comparison of reconstruction results of human tumor cell biospecimens. (a) LR FOV image of the sample. (a1) Enlarged region of interest. (b1)–(e1) Amplitude images reconstructed by the AA algorithm, SC-FPM, PC-FPM, and AA-C algorithm, respectively. (b2)–(e2) The enlargements of the region of interest of the amplitude images reconstructed by the different algorithms, respectively. (b3)–(e3) Phase images reconstructed by the AA algorithm, SC-FPM, PC-FPM, and AA-C algorithm, respectively. (b4)–(e4) The enlargements of the region of interest of the phase images reconstructed by the different algorithms, respectively.

The results of the reconstruction of human tumor cells using different algorithms are shown in Fig. 5. Figure 5(a) shows the LR FOV image of the specimen, and Fig. 5(a1) shows the corresponding enlarged region of interest (770×770  pixels). The reconstruction results for the AA algorithm are shown in Figs. 5(b1)–5(b4). Both the amplitude and phase images reconstructed without correction for LED position misalignments showed more streaks and folds, which significantly affected the reconstruction quality of the images. The reconstructed results of the SC-FPM are shown in Figs. 5(c1)–5(c4). We can see that the reconstructed results are significantly improved, with a significant reduction in streaks and folds; however, there are still some distortions and artifacts in the reconstructed phase image. For PC-FPM, the reconstructed phase image is significantly improved compared to Figs. 5(b3) and 5(c3), with a significant reduction in folds and artifacts; however, the amplitude image shows unexpected streaks, as shown in Figs. 5(d1)–5(d4). Finally, Figs. 5(e1)–5(e4) show the reconstruction results for the AA-C algorithm. In comparison to the first three algorithms, the overall clarity and smoothness of the reconstructed amplitude image are improved, and there are no stripes or artifacts in the phase image. The contours and details of the cells are clearer, and the reconstructed images have the best recovery effect, further validating the robustness of the AA-C algorithm.

**Fig. 6:**
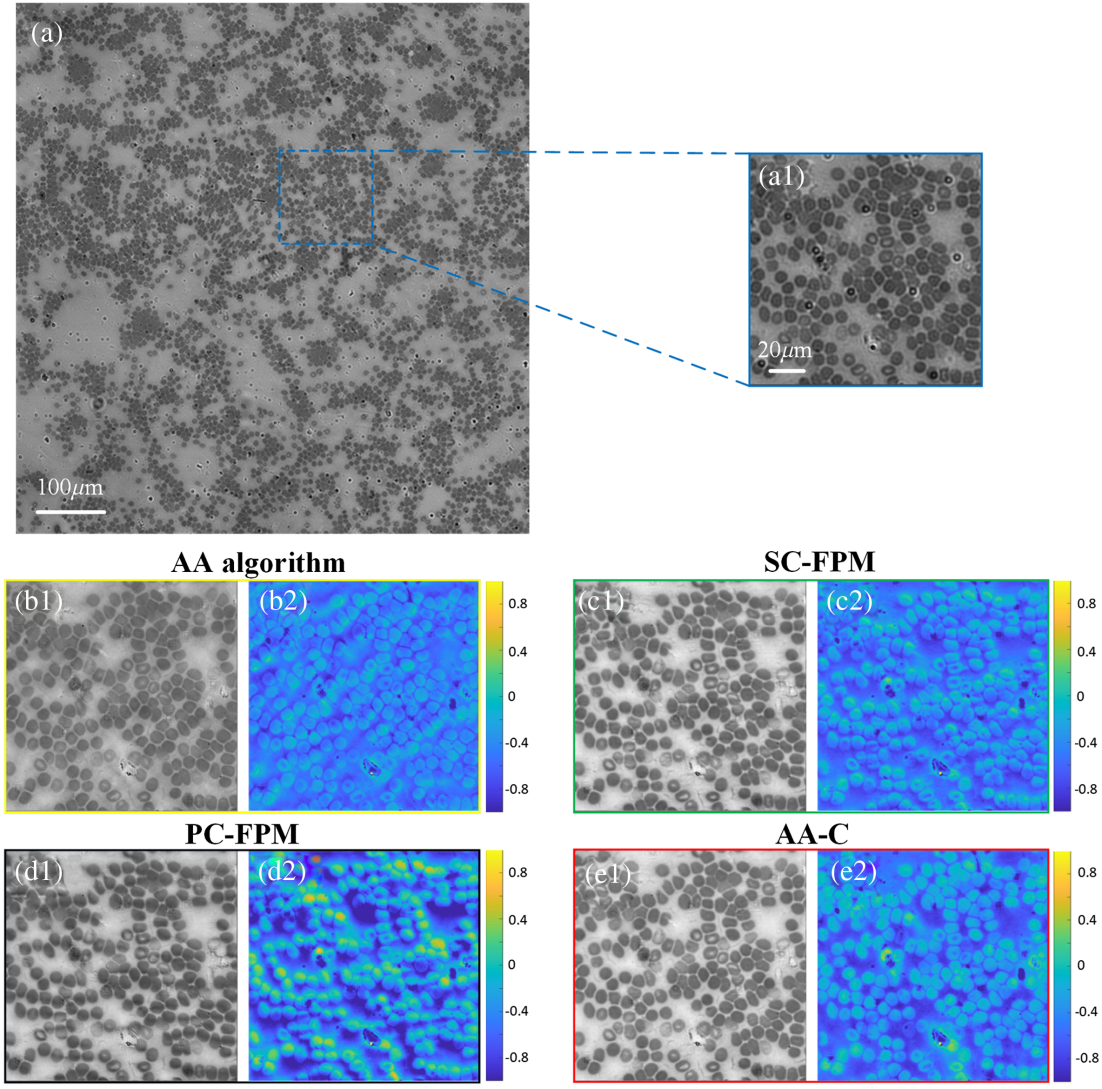
Comparison of experimental results of human blood smear sample. (a) LR FOV image of the sample. (a1) Enlarged region of interest. (b1)–(b2) Reconstruction results of the AA algorithm. (c1)–(c2) Reconstruction results of the SC-FPM algorithm. (d1)–(d2) Reconstruction results of the PC-FPM algorithm. (e1)–(e2) Reconstruction results of the AA-C algorithm.

Next, we selected human blood smears as samples for the experiment. A 5×5 LED array placed 50 mm beneath the sample. The spacing between neighboring LEDs was 5 mm, the wavelength of the LEDs was 531 nm, ${\mathrm{NA}}_{\mathrm{obj}}$ was 0.25, and the magnification was 10× and the pixel size of the sensor is 3.1  μm×3.1  μm. In all, 25 LR images were captured for FPM reconstruction during the experiment. The reconstruction results of human blood smears using different algorithms are shown in Fig. 6. Figure 6(a) shows the LR FOV image of a human blood smear specimen captured by the camera. Figure 6(a1) shows the magnified area (450×450  pixels) marked in (a). Figures 6(b1) and 6(b2) show the reconstruction results of the AA algorithm, where significant artifacts and stripes can be observed in both the reconstructed amplitude and phase images. As shown in Figs. 6(c1) and 6(c2), the amplitude image reconstructed with SC-FPM is greatly improved, but the phase image still has some artifacts and low contrast. The reconstruction results of the PC-FPM are shown in Figs. 6(d1) and 6(d2). When the position error of the LED array is large, the PC-FPM correction easily falls into the local optimal solution. This results in a decline in the correction ability of the position misalignment of the LED array, unsatisfactory quality of the reconstructed images, obvious artifacts in the amplitude image, and distortion and crosstalk in the phase image. Figures 6(e1) and 6(e2) show the reconstructed results of the AA-C algorithm, which effectively eliminated the artifacts. The reconstructed images are improved, the amplitude image background is clear and uniform, the phase image contrast is high, and there is no image distortion or obvious crosstalk.

Table 2 also compares the runtime and final iteration error for different correction algorithms in different biological specimen experiments. The results demonstrate that the proposed AA-C algorithm reconstructs the image with a smaller final iteration error requiring less runtime, which is faster compared to the SC-FPM and PC-FPM position correction algorithms.

**Table 2:** Comparison of running times and final iteration error for different correction algorithms in biological sample experiments. Bold characters represent the indicators of the proposed method are better.

Experiment	Algorithm	Time (s)	Final error ($10^{3}$)
Human tumor cell	SC-FPM	334.596	2.2857
	PC-FPM	527.714	1.9484
	AA-C	**298.623**	**1.3862**
Blood smear	SC-FPM	45.762	3.5382
	PC-FPM	59.931	4.4341
	AA-C	**41.196**	**2.8685**

## 4 Conclusion

This study presents improvements to the FPM correction method based on the SA algorithm and proposes a position misalignment correction method (AA-C algorithm) with an improved phase recovery strategy that effectively improves the image reconstruction quality by adding an adaptive control factor and optimizing the spectrum update strategy during the reconstruction process, which does not add to the complexities of the calculation and also has a robust convergence performance. Through simulations and using the USAF resolution target, human tumor cells, and blood smears as samples for the experiment, the effectiveness and robustness of the proposed AA-C algorithm for LED array position misalignment correction were verified. The proposed method accelerated the convergence speed and reduced the effect of LED array position misalignment on the FPM reconstruction quality. Compared with other advanced position correction methods, it can obtain a better reconstruction effect and improve the robustness of the FPM system. In addition, because the imaging efficiency of the FPM requires further improvement, we can combine LED multiplexing to further improve the imaging efficiency of the FPM and achieve faster and higher-quality high-throughput quantitative phase imaging. This will be the direction of our work in the future.

## Data Availability

Data that support the findings of this article are not publicly available at this time but the code can be obtained from the authors upon reasonable request.
